# Phylogenetic and DNA methylation analysis reveal novel regions of variable methylation in the mouse IAP class of transposons

**DOI:** 10.1186/1471-2164-14-48

**Published:** 2013-01-23

**Authors:** Christopher Faulk, Amanda Barks, Dana C Dolinoy

**Affiliations:** 1 Department of Environmental Health Sciences, University of Michigan, 1415 Washington Heights, Ann Arbor, MI 48109-2029, USA

**Keywords:** Epigenetics, DNA methylation, Metastable epiallele, Phylogeny, Transposon, Mouse

## Abstract

**Background:**

Select retrotransposons in the long terminal repeat (LTR) class exhibit interindividual variation in DNA methylation that is altered by developmental environmental exposures. Yet, neither the full extent of variability at these “metastable epialleles,” nor the phylogenetic relationship underlying variable elements is well understood. The murine metastable epialleles, A^vy^ and Cabp^IAP^, result from independent insertions of an intracisternal A particle (IAP) mobile element, and exhibit remarkably similar sequence identity (98.5%).

**Results:**

Utilizing the C57BL/6 genome we identified 10802 IAP LTRs overall and a subset of 1388 in a family that includes A^vy^ and Cabp^IAP^. Phylogenetic analysis revealed two duplication and divergence events subdividing this family into three clades. To characterize interindividual variation across clades, liver DNA from 17 isogenic mice was subjected to combined bisulfite and restriction analysis (CoBRA) for 21 separate LTR transposons (7 per clade). The lowest and highest mean methylation values were 59% and 88% respectively, while methylation levels at individual LTRs varied widely, ranging from 9% to 34%. The clade with the most conserved elements had significantly higher mean methylation across LTRs than either of the two diverged clades (p = 0.040 and p = 0.017). Within each mouse, average methylation across all LTRs was not significantly different (71%-74%, p > 0.99).

**Conclusions:**

Combined phylogenetic and DNA methylation analysis allows for the identification of novel regions of variable methylation. This approach increases the number of known metastable epialleles in the mouse, which can serve as biomarkers for environmental modifications to the epigenome.

## Background

Across mammalian genomes, the retrotransposon class of repeat elements make up nearly half the nuclear DNA, with long terminal repeats (LTRs) alone accounting for 8-10% [1]. Many retrotransposons retain the ability to move (transpose) to new locations in the genome, a potentially deleterious phenomenon that can result in direct disruption to coding regions [2,3]. Therefore, the suppression of retrotransposons is vital to organismal survival, particularly through sensitive life stages, and is primarily accomplished via epigenetic mechanisms, including DNA methylation [4,5]. Epigenetic suppression is typically established early in development at the blastocyst stage and maintained during differentiation of the primordial germ cells [6]. Importantly, in certain cases suppression is incomplete, leading to adverse consequences [7]. Alternatively, this incomplete suppression may be an evolutionarily selected method of fine-tuning gene expression [8]. Genome-wide, little is known about the extent to which interindividual variation of DNA methylation at these elements is affected by environmental factors and/or their phylogenetic lineage [9].

Intracisternal A particles (IAP) are a class of murine retrotransposons named for the appearance of budding doughnut shaped particles in the cisternae of the endoplasmic reticulum [10]. IAPs are members of the endogenous retrovirus family (ERV) class II [11]. Structurally, a full length IAP consists of *gag*, *pro*, and *pol* genes capped at each end by a direct pair of LTRs, each approximately 350 bp in length [12]. However, the largest majority of IAP transcripts issue from truncated 5.4 kb copies of IAP of subtype IΔ1 rather than full length 7.2 kb copies [13]. Previous work has identified only a subset of elements likely to be active, due to genomic position, sequence mutation, or methylation status [13]. The number of paralogous IAP LTRs in the genome has been uncertain with estimates ranging from less than 1000 to over 9000 with significant polymorphism among strains [10,14,15]. This family of ERVs is highly active and rapidly transposing, with estimates of 60% of elements being strain specific [15]. It is advantageous for these mobile elements to be kept under tight control by heavy DNA methylation and tight chromatin structure.

A small number of “metastable epialleles” have been identified in mice (e.g. A^vy^, Cabp^IAP^, and Axin^Fu^) in which variable methylation of the 5′ LTR of an antisense IAP insertion results in dysregulation of gene expression concomitant with the level of methylation [16-20]. Metastable epialleles show variable expression among individuals with epigenetic profiles that are consistent across tissues and are thus likely to have been set prior to germ line differentiation [21-23]. Further, the distribution of variable expressivity has been shifted at these metastable epialleles following maternal exposure to nutritional and environmental factors [21,22,24,25]. To date, no studies have determined the genetic relationship underlying the ability of these IAP LTRs to be variably methylated. Two of the epialleles, A^vy^ and Axin^Fu^, were identified due to dramatic phenotypes induced by their insertion into nearby protein-coding genes [18,26]. Conversely, Cabp^IAP^ was identified by a search of Genbank cDNA databases for chimeric sequence containing IAP LTR and genic sequence [20].

To systematically investigate the relationship of genetics and metastability we applied phylogenetic methods to identify candidate IAP retrotransposons and validate their variable methylation status. Through this approach, we find the phylogeny of metastable loci sheds light on the evolutionary basis of metastability. Additionally, the validated analysis of epigenetic state across individual isogenic mice now increases the number of known metastable epialleles, which can serve as a test panel for environmental modifications to the epigenome.

## Results

### IAP distribution in the genome

**Figure 1:**
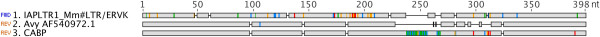
**Multiple sequence alignment. **The sequence of the A^vy ^and Cabp^IAP^ LTR insertions are compared to the IAPLTR1_Mm consensus sequence. A^vy^ shares 98.5% sequence identity with Cabp^IAP ^(85% sequence identity over the length of Cabp^IAP^). Colors indicate base changes from consensus (A = red, T = blue, C = yellow, G = green).

Initial pairwise alignment of the two most well-studied metastable epialleles, A^vy^ and Cabp^IAP^ revealed that the LTRs of these two insertions exhibit 98.5% sequence identity over the length of shared sequence (Figure 1). RepeatMasker was used to scan the C57BL/6 (mm9) mouse genome and identified 10802 IAP LTR elements. The majority of these LTRs were pairs found on the 5’ and 3’ end of the same IAP insertion, however a fraction were present as solo LTRs. Since the IAPs underlying both A^vy^ and Cabp^IAP^ are of the class IΔ1, type IAPLTR1_Mm as identified in RepBase, this subtype was used to filter the complete list of transposons, resulting in a total list of 1388 IAPLTR1_Mm elements after removing 65 unmapped, short, or non-alignable elements. There was no bias in the orientation of insertion as 691 sense and 707 anti-sense IAPLTR1_Mm elements were identified throughout the genome.

### Phylogenetic similarity of known metastable IAPs

**Figure 2:**
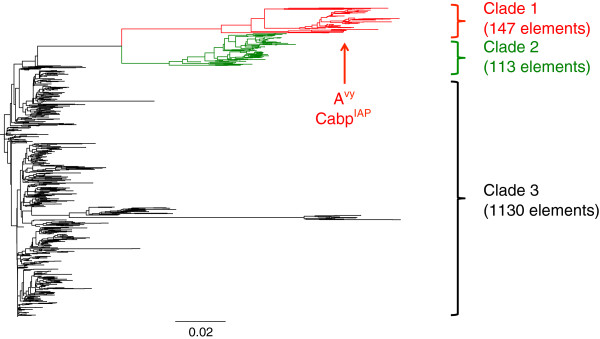
**Neighbor-joining tree. **Illustrated are 1388 IAPLTR_Mm elements drawn from the C57BL/6 genome and including A^vy ^and Cabp^IAP ^elements for a total of 1390. Subclades are highlighted in red (clade 1) and green (clade 2), with the remaining elements in black (clade 3). The bifurcation of subclades 1 and 2 demonstrates duplication and divergence events followed by rapid radiation of these subfamilies. Scale bar indicates number of nucleotide substitutions per site.

**Figure 3:**
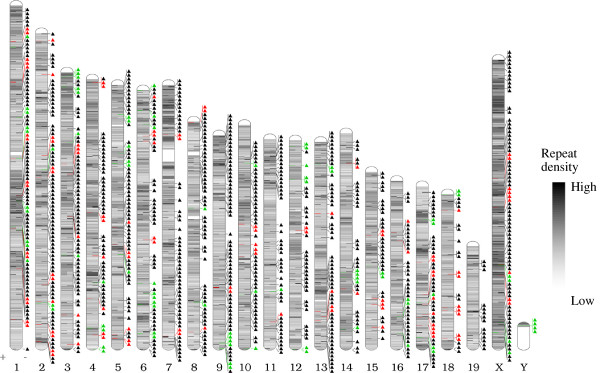
**IAP element insertions. **The 1388 insertions of IAPLTR1_Mm are plotted against their genomic location. The color of the clade corresponding to the insertion’s position on the phylogenetic tree is highlighted to the right of each chromosome. Elements from each clade in the tree are found dispersed throughout the genome. The color scale internal to the chromosomes corresponds to overall repetitive element density.

A neighbor-joining tree of these 1388 elements revealed three distinct clades with A^vy^ and Cabp^IAP^ clustering within clade 1 (red), more closely than 99% of all other elements (Figure 2). The largest cluster, clade 3 (black), contains the most conserved elements, and consists of 1130 sequences with an average sequence divergence of only 1.8% from the consensus. Clade 1 (red) and clade 2 (green) contain 147 and 113 elements each, and are more divergent, with 9.1% and 12.9% average differences from the consensus respectively. Bias in location of these insertions was not identified, with members of each clade represented across numerous chromosomes and locations (Figure 3). Unique identifiers were assigned to each element in our list, in the format IAP27-IAP1414, used throughout the study.

### Characterization of interindividual metastability

**Figure 4:**
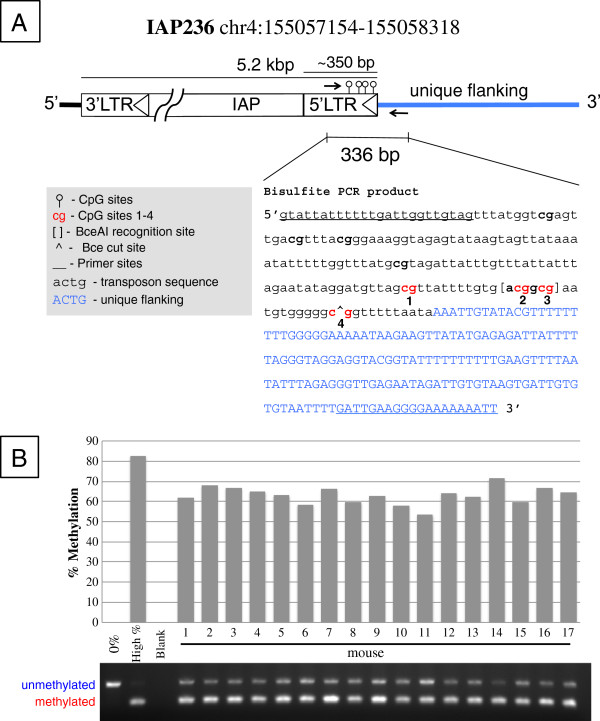
**CoBRA design. **For each candidate locus we amplify a portion of the antisense strand of the 5′ LTR and unique flanking sequence (arrows) containing a BceAI recognition site. (**A**) The product of IAP236 is shown with cut sites and CpGs indicated by numbers 1–4. Sites 2 and 3 are conserved in all candidate loci and cut. PCR primers are underlined. (**B**) Percent methylation is derived from relative intensity of the cut vs. uncut bands. This locus exhibits an 18% range in variable methylation. Controls are shown in lanes 1 and 2, indicating the specificity and completeness of the digestion.

From these candidates 7 from each clade were randomly selected to test for variable methylation using combined bisulfite and restriction analysis (CoBRA). Primers for CoBRA were designed with the forward primer internal to the IAP sequence and the reverse primer located in the flanking sequence (Figure 4A), providing an amplicon specific to an insertion and containing homologous IAP element sequence (Table 1). BceAI enzyme was chosen since each element contains two CpG sites as part of a BceAI restriction site, ACGGCG. Loss of methylation at either or both would prevent BceAI activity. Consequently, the relative intensity of cut vs. uncut bands gives a semi-quantitative measurement of methylation at these sites (Figure 4A). Uncut bands correspond to unmethylated DNA while cut bands correspond to methylated DNA. The two CpG sites examined here exhibited interindividual variation in previous studies from our group [24,27], and are identified as sites 2 and 3 in this study. A quantitated CoBRA gel image of IAP236 is depicted in Figure 4B, revealing an 18% range of methylation at the cut site.

**Table 1:** PCR primers and conditions for CoBRA analysis

**IAP**	**Clade**	**Position**	**Forward primer**	**Reverse primer**	**Nearest gene**	**Distance**	**Cycles**	**Temperature (°C)**
CabpIAP	1 (red)	chr2:154179911-154180159	ATTATTTTTTGATTGGTTGTAGTTTATGG	CACCAACATACAATTAACA	Cdk5rap1	intron	39	47
iap31	1 (red)	chr1:89356306-89357454	TAAGAAGTAAGAGAGAGAAGTAA	CCAAACAAATCCAAAAACCTAA	-	-	45	52
iap44	1 (red)	chr10:77413077-77414228	GGTTAGGAAGAATATAATAATTAG	CTAAAAATAAAACCCAAAAACCC	Trpm2	Intron	45	52
iap51	1 (red)	chr12:53835543-53836696	GGGAAAAATAGAGTATAAGTAG	TCTAACAACTACCCACAAAAAAT	Akap6	Intron	40	52
iap77	1 (red)	chr17:64098002-64099153	AAGTAAGAGAGAGAGAAAAT	TATAACCCCCAAATAACTAACAT	-	-	45	52
iap90	1 (red)	chr18:47812857-47814008	GGGAAAAATAGAGTATAAGY	CACTAAAAACAACAATCTAACAAC	Gm5095	Intron	43	52
iap110	1 (red)	chr2:72112889-72114056	AAGTAAGAGAGAGTAAGAAGTAA	CATATACAACACTTAAAACAAAACC	Rapgef4	18 kb upstream	45	52
iap176	2 (green)	chr1:127212941-127214106	TTTATATTTTTGGGAGTTAGG	AACACTCTTCTACAATAACATCT	-	-	40	52
iap182	2 (green)	chr1:26288894-26290059	TGTTTATATTTTTGGGAGTTAG	AACCTACTTCATCTTAAAAC	-	-	45	52
iap186	2 (green)	chr10:24567718-24568883	GTATTATTTTTTGATTGGTTGTAG	AAACCCACTAATTCTTCCTAT	Enpp3	12 kb upstream	40	52
iap195	2 (green)	chr12:25079835-25080998	TTGTTTATATTTTTGGGAGT	CACCTTATATTCTCCAAAAAAAC	-	-	40	52
iap236	2 (green)	chr4:155057154-155058318	GTATTATTTTTTGATTGGTTGTAG	AATTTTTTTCCCCTTCAATC	Mib2	15 kb upstream	45	52
iap268	2 (green)	chr9:123106561-123107725	TTTATATTTTTGGGAGTTAGG	ACACCTAACATCATCTAAAT	Cdcp1	Intron	45	52
iap281y	2 (green)	chrY:2136760-2137925	GGTTAGGAAGAATATTATAGA	TACACCAAAAACAAACCAAA	Rbmy1a1	8 kb downstream	45	52
iap506	3 (black)	chr12:74416066-74417247	AGTAAGAAGTAAGAGAGTAAGAA	CTACACCCCAAAAATAATAAAAAC	Slc38a6	Intron	45	52
iap655	3 (black)	chr15:11992027-11993248	AGAGAAAAGTAAGAGAGAGAAAA	AAAACAAAAAAAACTACACCC	-	-	45	52
iap1112	3 (black)	chr6:101092968-101094168	TAAGAGAGAGAGAAAAGTAAGAGA	CCACCAAAATAAAAACTCAAAAC	Pdzrn3	6 kb downstream	43	52
iap1248	3 (black)	chr8:63849572-63850723	TTTTTAGGAGTTAGAGTGTA	CTCCTTTCTAATTTTATTCTCCA	Sh3rf1	Intron	45	53
iap1252	3 (black)	chr8:47435363-47436559	AGAAAAAGTAAGAGAGAGAGAAA	AACCCTAAAATTCCTCAAAAAAC	Helt	56 kb upstream	40	54
iap1259	3 (black)	chr8:8319882-8321071	AAGAAGTAAGAGAGTAAGAAGTAA	ACAAAAAATCAACTAAACTCTAC	-	-	45	53
iap1334	3 (black)	chr9:121236806-121238001	AAGTAAGAGAGAGAGAAAAGTAA	RACTACTACTAAAAACCCACAA	Trak1	Intron	40	54

**Figure 5:**
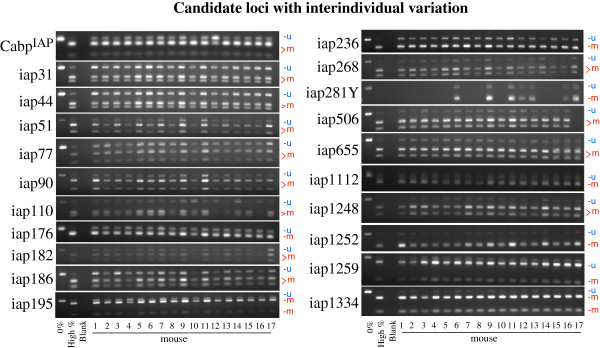
**Candidate loci with interindividual variation. **DNA from 17 isogenic mouse livers at d21 was isolated and bisulfite converted. Amplification and digestion of 21 representative IAP elements show variable levels of methylation per locus and mouse. Upper bands indicate uncut PCR product corresponding to the unmethylated BceAI site. Lower bands indicate a fully methylated BceAI site consisting of CpGs 2 and 3 from Figure 4. Locus 281Y is located on the Y chromosome and is therefore amplified only from male animals.

A group of 17 isogenic A^vy^/a genotype mice of both genders (N = 7 males and N = 10 females) from 7 litters was sacrificed at weaning and DNA was extracted from the liver. Following bisulfite conversion, candidate regions were amplified via PCR and digested with BceAI (Figure 5). A total of 21 representative loci, 7 from the red clade including Cabp^IAP^, and 7 each from the green and black clades were evaluated. The 0% control (lane 1) was uncut in all loci, while the high percent methylated control was nearly completely digested in all loci (lane 2) indicating sufficient enzyme activity and specificity.

**Figure 6:**
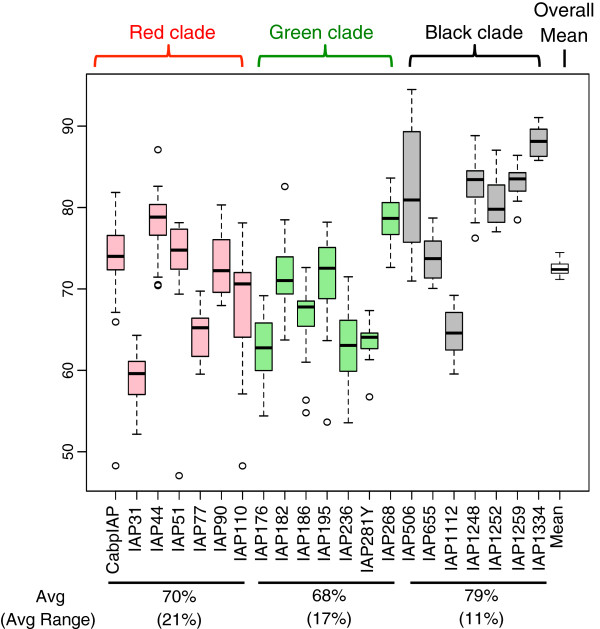
**Boxplot of methylation values. **Interindividual variation in each IAP correlates with phylogenetic clade. Average methylation values are lower for red and green clades (70% and 68%) than for the black clade (79%) suggesting higher methylation for older insertions. The average range per clade, in parentheses, is also lower, however the standard deviation is similar across clades.

The mean methylation across all mice for all IAP insertions was 72.5% and no mouse deviated by more than 3% (range 71-74%), showing no statistical difference (p > 0.99) (Table 2). Specific loci however, exhibited much greater variability. We found that mean methylation of individual IAPs fluctuated from a low of 59% to a high of 88% and standard deviations varied from 1.9 to 7.3, indicating that individual transposons can differ dramatically in both average value and standard deviation among mice. Thus, methylation variability ranged from 9% for the least variable element to greater than 34% for the most variable element. There were no significant differences in methylation between sexes. Importantly, the average methylation was significantly lower for both red clade elements, 70% (p = 0.017), and green clade elements, 68% (p = 0.040) than for black clade elements, 79%. Interestingly, the range through which a particular element can be methylated is also correlated with its position within the phylogenetic tree (Figure 6). The average range for red clade elements is 21%, 17% for green clade elements, and just 11% for black clade elements. The element with the greatest range is in the red clade (Cabp^IAP^) while the element with the greatest dispersion as measured by interquartile range is found in the black clade (IAP506).

**Table 2:** CoBRA quantitated percent methylation across 17 mice and 21 IAP elements

	**Red clade**							**Green clade**							**Black clade**							
	**CabpIAP**	**IAP31**	**IAP44**	**IAP51**	**IAP77**	**IAP90**	**IAP110**	**IAP176**	**IAP182**	**IAP186**	**IAP195**	**IAP236**	*IAP281Y	**IAP268**	**IAP506**	**IAP655**	**IAP1112**	**IAP1248**	**IAP1252**	**IAP1259**	**IAP1334**	**Average**
Mouse 1	74	52	70	77	60	80	71	69	70	61	70	62		77	77	76	66	LOD	83	82	88	72
Mouse 2	77	60	77	75	60	71	72	60	71	68	76	68		84	86	70	69	83	77	78	86	73
Mouse 3	81	57	77	78	66	76	72	66	74	55	73	67		76	71	76	68	84	78	84	LOD	72
Mouse 4	72	61	80	77	65	68	69	56	77	56	54	65		73	82	70	60	89	80	81	86	71
Mouse 5	74	59	71	78	61	80	75	65	70	69	69	63		80	81	70	LOD	76	78	82	90	73
Mouse 6	76	57	78	74	65	73	71	63	64	67	76	58	64	77	94	76	63	78	LOD	85	86	72
Mouse 7	66	62	80	75	61	73	78	54	67	65	64	66		75	75	74	60	85	78	82	86	71
Mouse 8	76	60	75	78	62	72	64	63	71	65	73	60		78	93	70	62	81	79	84	LOD	72
Mouse 9	67	62	83	73	64	70	67	69	75	69	74	63	67	76	76	74	68	87	83	86	86	73
Mouse 10	82	62	80	47	68	69	57	57	78	73	65	58		82	92	79	60	84	82	81	91	72
Mouse 11	70	60	87	69	65	71	73	65	70	68	72	54	64	80	76	73	63	87	87	84	90	73
Mouse 12	48	61	77	70	66	71	57	63	74	71	76	64	61	84	94	75	64	81	82	81	89	72
Mouse 13	79	64	81	73	64	69	66	67	69	68	75	62	64	80	81	72	66	LOD	77	84	86	72
Mouse 14	79	58	79	72	68	76	57	63	69	68	78	71		79	82	77	69	84	84	84	90	74
Mouse 15	74	55	80	75	68	74	71	60	72	66	71	60		81	80	71	64	83	84	85	89	73
Mouse 16	73	56	81	71	70	69	48	60	83	71	75	67	57	83	74	73	66	82	79	85	88	72
Mouse 17	76	59	71	78	62	78	72	66	66	67	68	65	65	78	LOD	77	65	82	79	83	91	72
mean	73	59	78	73	64	73	67	63	72	66	71	63	63	79	82	74	65	83	81	83	88	72
median	74	60	79	75	65	72	71	63	71	68	73	63	64	79	81	74	65	83	80	84	88	72
stdev	8	3	4	7	3	4	8	4	5	5	6	4	3	3	8	3	3	3	3	2	2	1
min	48	52	70	47	60	68	48	54	64	55	54	54	57	73	71	70	60	76	77	78	86	71
max	82	64	87	78	70	80	78	69	83	73	78	71	67	84	94	79	69	89	87	86	91	74
range	34	12	17	31	10	12	30	15	19	18	25	18	11	11	24	9	10	13	10	8	5	3

* IAP located on the Y chromosome, only measured in male animals.

LOD = Limit of Detection.

## Discussion

Our bioinformatic analysis identified 10802 IAP LTRs, consistent with recent estimates of ~5400 IAP transposons in the mouse genome as the vast majority of detected insertions in our analysis were paired on either end of a single IAP insertion [15,28]. To validate candidate LTRs as metastable, we focused primarily on sequences of the specific subtype of IAP known in two cases to be variably methylated (IAPLTR1_Mm). Both the A^vy^ and Cabp^IAP^ epialleles exhibit variable DNA methylation at specific CpG sites, which can be influenced by developmental environmental exposure [24]. Phylogenetic analysis revealed that both epialleles clustered in one of two divergent subclades in the IAPLTR1_Mm family. Combined bisulfite and restriction analysis performed on 20 additional loci found interindividual methylation to be a common feature of all elements in this family. Though the degree of variation ranged from 9% to 34% among elements within each mouse, average methylation across all LTRs varied only 3%, a confirmation that global methylation of repetitive loci does not necessarily reflect locus specific deviation. Interestingly, on average, IAP methylation was higher and less variable in the more numerous, phylogenetically conserved elements (black clade 3). While the standard method of dating LTR insertions by measuring accumulated mutations in the 5′ and 3′ LTRs is inapplicable in this case because of their young age, the percent divergence from consensus strongly suggests the two derived subclades are younger. The increased methylation of the older IAPs here fits with the evidence of Reiss et al. who found a positive correlation of age with methylation in another family of ERVs, the Early Transposon (ETn) class and hypothesized this would be a general finding in ERVs [29].

Though retrotransposons, including IAPs, are generally highly methylated, important windows of vulnerability exist in which genomic methylation is globally reprogrammed [4,6,21]. Reactivation during genomic reprogramming is likely responsible for the frequent germ line insertion in this lineage of IAPs in rodents, counterbalanced by selective pressure to maintain epigenetic silencing in somatic cells [17]. The presence of two diverged clades in our phylogeny suggests that the ancestor of the A^vy^ and Cabp^IAP^ alleles at two times collected advantageous mutations that allowed it to escape genomic control and proliferate successfully throughout the genome following the master gene model [30]. Their genomic distribution also suggests that they retained retrotranspositional activity after divergence, a finding also supported previously [15].

Epigenomic studies are increasingly cataloging variation across tissues, disease states, ages, and environmental exposures. It is therefore important to understand the baseline naturally extant variation in a population of isogenic, unexposed, age-matched animals and to provide assays to detect shifts in variation mediated by other conditions. Determination of interindividual variation in DNA methylation has followed a number of methods, including coat color shifts in A^vy^ mice [27], bisulfite sequencing, northern blots for transcript quantity [31], methylation specific PCR, and pyrosequencing [32]. The method we use here, combined bisulfite and restriction analysis (CoBRA), exploits site-specific enzyme digestion to determine methylation status. Advantages of CoBRA include semi-quantitative analysis, and relatively inexpensive costs, thereby facilitating the high throughput necessary for epidemiological testing. Indeed, CoBRA has been used to assess the epigenotype of colorectal tumors, providing direct health implications [33]. Additionally, automation platforms such as the QIAxel system (Qiagen Inc., Valencia, CA) would increase the speed of sample quantitation after enzyme digestion. Our candidate loci were also mined from the C57BL/6 genome, and as such, they are more likely to be useful as epigenetic markers in both mutant and exposed mice than previous epialleles (A^vy^ and Axin^Fu^), which are restricted to specific lines.

Previous studies utilizing non-phylogenetic methods of variably methylated candidate identification have identified retroelements co-located with active histone modification (H3K4me3) [34]. Further, IAPs, unlike other transposons, can induce changes in nearby chromatin marks, but no phylogenetic basis underlying epigenetic states has been explored [2]. The close sequence identity of A^vy^ and Cabp^IAP^ insertions may be associated structurally with directed epigenetic modifications by targeting enhanced recruitment of *trans* factors governing their metastability. Alternatively, high sequence similarity in the divergent clades may have resulted from very recent mobilization and high activity of the parent element and its rapid proliferation. We speculate that variable DNA methylation, being a general property of elements we tested from these clades, explains both the success of this lineage in duplication, and their interindividual variation. This hypothesis is borne out by the average methylation being higher in the black clade, containing the largest number, most conserved, and likely oldest, elements. Though in general, new insertions are considered deleterious, some have been co-opted by the genome as a mechanism of driving gene expression, and epigenetically labile insertions may be under selection to “fine tune” that expression [35]. Our analysis supports the notion that mutation to escape genomic control is correlated with both increased retrotranspositional activity and decreased methylation. Consequently, these data are a strong indication that interindividual variation in methylation at repetitive elements is the default condition, rather than a rare occurrence, and that shifts in methylation profiles could affect numerous transcripts in the genome. Thus, environmental exposures capable of altering the epigenome could potentially have widespread effects for gene regulation.

## Conclusions

Combined phylogenetic and DNA methylation analysis resulted in the identification of novel regions of variable methylation. The phylogenetic analysis revealed two duplication and divergence events subdividing this family into three clades. Importantly, average methylation was significantly lower for both the red and green clade elements, compared to black clade elements, supporting the notion that phylogenetically older and more uniform elements tend to be more highly methylated than younger and diverged elements. Thus, through this endeavor the number of known metastable epialleles in the mouse has dramatically increased. These sites can now serve as biomarkers for environmental modifications to the epigenome. Parallel approaches in humans are underway.

## Methods

### Computational analysis

For the A^vy^ allele, the LTR studied was obtained from Genbank accession number [GenBank:AF540972.1], position 10–345. For Cabp^IAP^, the LTR sequence was obtained from [GenBank:AL732601.16], position 179834–180227. For CoBRA and pyrosequencing analysis, four CpG sites were referenced from the A^vy^ allele coordinates, [GenBank:AF540971.1], positions 365, 378, 380, and 393. These are annotated as positions 1–4 in this report.

Mouse genome version 37.61(mm9, 2007) was downloaded from NCBI and scanned with a local installation of RepeatMasker version open-3.3.0 http://www.repeatmasker.org using the RMblast engine http://www.repeatmasker.org/RMBlast.html with the following parameters, “RepeatMasker -xsmall -s -species mouse”. The repeat database was obtained from RepBase release 20090604 http://www.girinst.org[36]. The output was filtered for elements annotated as IAPLTR and counted to 10802. Since each IAP element contains two LTRs, this represents approximately 5000 full-length IAP elements of all subtypes. We next filtered for elements of type IΔ1, subtype IAPLTR1_Mm and removed non-chromosomal elements and 40 short elements below 330 bp. The resulting dataset contained 707 elements on the sense strand and 691 on the complementary strand (Additional file 1: Table S1). A further 10 elements were removed for lack of alignment to the IAPLTR1_Mm consensus. Alignment was performed by Geneious software version 5.6.5 http://www.geneious.com/ using the Geneious alignment algorithm with default parameters (cost matrix = 65% similarity, gap open penalty = 12, gap extension penalty = 3). The phylogenetic tree was built using neighbor-joining, no outgroup, Jukes-Cantor genetic distance model. Bootstrap of 1000 iterations showed no substantial differences in the divergence of the major clades (data not shown).

Genomic sequence for each element (for alignment and tree building) and 400 bp of flanking sequence (for primer design) was obtained by submission of the RepeatMasker generated coordinates to the Galaxy Project https://main.g2.bx.psu.edu/[37]. Unique numbers were assigned to each IAP here to aid in reference and used throughout the study (Additional file 1: Table S1). A second alignment was performed with flanking sequence to select all sites with 5’ LTR sequence, complete IAP structure, and 3’ LTR sequence. Removal of duplicate entries that matched to the 3’ LTR copy and other LTRs with repetitive flanking sequence resulted in a total of 366 candidate elements for CoBRA [38,39]. All primers were designed to amplify the antisense strand of the 5’ LTR and proximal flanking sequence (Table 1).

### DNA extraction and conversion

Mice were obtained from a forced heterozygous colony carrying the A^vy^ allele that has been maintained with sibling mating for over 220 generations, resulting in a genetically invariant background initially based upon the C3H strain [21]. Virgin wild-type dams, 6 weeks of age, were fed phytoestrogen free AIN-93 G diets (diet 95092 with 7% corn oil substituted for 7% soybean oil; Harlan Teklad, Madison, WI) and were mated. A^vy^/a offspring were sacrificed at d21 and livers flash frozen for DNA extraction. A total of 17 animals (N = 7 males and N = 10 females) and some sets of littermates were used for this study. DNA extraction was performed using a standard Phenol-Chloroform protocol. Using the Qiagen Epitect kit automated on the Qiagen QIAcube® purification system, approximately 1 μg of genomic DNA was treated with sodium bisulfite. The treatment converts unmethylated cytosines to uracil, read as thymine during polymerase chain reaction (PCR), whereas the methylated cytosines are protected and remain unconverted [40].

Animals used in this study were maintained in accordance with the *Guidelines for the Care and Use of Laboratory Animals* (Institute of Laboratory Animal Resources, 1996) and were treated humanely and with regard for alleviation of suffering. The study protocol was approved by the University of Michigan Committee on Use and Care of Animals.

### Combined bisulfite restriction analysis

CoBRA [39] was performed with enzyme BceAI (New England Biolabs®) using the following conditions for PCR: 50 ng DNA, 0.5 μl each forward and reverse primer at 10pM/μl concentration, 15 μl HotStarTaq master mix (Qiagen Inc., Valencia, CA), 3 μl Rediload™ (Invitrogen Inc., Grand Island, NY), 10 μl water for a total of 30 μl reaction volume. The reaction was run at 52°C annealing temperature for 45 cycles on a C1000 Bio-Rad thermal cycler pcr machine (Hercules, CA). Some reactions were run with altered conditions (Table 1). Enzyme digestion was performed according to manufacturer protocol, briefly, 10 μl of PCR product, 3 μl BSA (10×), 3 μl Rediload™, 3 μl NEB buffer 3 (10×), 1 μl BceAI enzyme (1 Unit), and 10 μl of water were combined for a 30 μl reaction and incubated at 37°C for 8 hours, followed by 65°C for 20 minutes and run out on a 2% agarose gel. Gels were visualized on a Gel Doc XR™ by Bio-Rad. Image quantitation was performed with Quantity One version 4.6.7 using default options (all lanes, auto detect = yes, normalize = yes, sensitivity = 10, size scale = 5) and reporting relative quantity of each band per lane. Lanes with top bands too faint for detection were considered below the limit of detection (LOD) and removed from further analysis (marked as blank in Table 2). Statistical analysis to determine significance of clade methylation differences was performed using SPSS (IBM, New York, NY) by ANOVA with multiple comparisons Tukey adjustment.

Control DNA was generated in-house by whole genome amplification using the GenomePlex® kit (Sigma-Aldrich, St. Louis, MO) for 0% methylated DNA and by CpG methylase, M.SssI treatment (Zymo Research, Irvine CA) for highly methylated control, both according to manufacturer protocols.

## Abbreviations

IAP: Intracisternal A particle; LTR: Long terminal repeat; A^vy^: Viable yellow agouti; Cabp^IAP^: CDK5 activator binding protein, IAP insertion; CoBRA: Combined bisulfite and restriction analysis; PCR: Polymerase chain reaction; LOD: Limit of detection.

## Competing interests

The authors declare no competing interests.

## Authors’ contributions

CF and DCD conceived the study. CF carried out the repeat masking, sequence alignment, and tree building. CF and AB performed the DNA extraction, conversion, primer design, and CoBRA testing. CF, AB, and DCD performed data analysis. CF and AB were responsible for animal husbandry. CF and AB drafted the manuscript and created the figures with editorial guidance from DCD. All authors read and approved the final manuscript.

## Supplementary Material

Additional file 1 **Table S1. **IAPLTR1_Mm elements derived from the mm9 genome. Click here for file
